# Macronutrient Determinants of Obesity, Insulin Resistance and Metabolic Health

**DOI:** 10.3390/biology10040336

**Published:** 2021-04-16

**Authors:** Jibran A. Wali, Samantha M. Solon-Biet, Therese Freire, Amanda E. Brandon

**Affiliations:** 1 Charles Perkins Centre, University of Sydney, Sydney, NSW 2006, Australia; jibran.wali@sydney.edu.au (J.A.W.); samantha.biet@sydney.edu.au (S.M.S.-B.); therese.freire@sydney.edu.au (T.F.); 2 School of Life and Environmental Sciences, Faculty of Science, University of Sydney, Sydney, NSW 2006, Australia; 3 School of Medical Sciences, Faculty of Medicine and Health, University of Sydney, Sydney, NSW 2006, Australia

**Keywords:** insulin resistance, macronutrients, obesity

## Abstract

**Simple Summary:**

Worldwide, overweight and obesity are an ever-increasing problem. Insulin resistance is often associated with obesity and is a precursor to a range of other diseases (e.g., cardiovascular disease and type 2 diabetes). In this review, we discuss the role of dietary carbohydrates, fats, and proteins in metabolic health. We also review how the “one nutrient at a time” approach of traditional research may not be the most appropriate way, and how the use of the geometric framework for nutrition platform could assist in reconciling apparently contradictory findings in the literature.

**Abstract:**

Obesity caused by the overconsumption of calories has increased to epidemic proportions. Insulin resistance is often associated with an increased adiposity and is a precipitating factor in the development of cardiovascular disease, type 2 diabetes, and altered metabolic health. Of the various factors contributing to metabolic impairments, nutrition is the major modifiable factor that can be targeted to counter the rising prevalence of obesity and metabolic diseases. However, the macronutrient composition of a nutritionally balanced “healthy diet” are unclear, and so far, no tested dietary intervention has been successful in achieving long-term compliance and reductions in body weight and associated beneficial health outcomes. In the current review, we briefly describe the role of the three major macronutrients, carbohydrates, fats, and proteins, and their role in metabolic health, and provide mechanistic insights. We also discuss how an integrated multi-dimensional approach to nutritional science could help in reconciling apparently conflicting findings.

## 1. Introduction

Obesity and associated metabolic diseases such as type 2 diabetes have become global epidemics with more than 600 million obese and more than 450 million diabetic adults worldwide [1,2]. Of the various factors contributing to metabolic impairments, nutrition is the major modifiable factor that can be targeted to counter the rising prevalence of obesity and metabolic diseases. However, despite decades of research, the specifics of a nutritionally balanced “healthy diet” are unclear and none of the dietary interventions have been successful in achieving long-term population-wide compliance or a significant and sustained reduction in body weight [3,4]. Protein, fat and carbohydrate are the major dietary macronutrients [4]. The World Health Organisation (WHO) recommends consuming 55–75% of daily energy from carbohydrate, 15–30% energy from fat, and 10–15% energy from protein [4], while the acceptable macronutrient distribution range (AMDR) for the United States recommends 10–35% daily energy from protein, 20–35% from fat and 45–65% from carbohydrate [5]. Interestingly, all three macronutrients (or at least certain sub-types) have been linked to insulin resistance and diabetes [6,7,8,9,10,11,12,13,14,15,16]. In this narrative review, we provide a brief overview of the potential role of protein, fat and carbohydrate intake in obesity and metabolic disorders. We also discuss the mechanisms that may link the consumption of these macronutrients to insulin resistance. Finally, we explain how an integrated multi-dimensional approach to nutrition science could help in reconciling apparently conflicting findings.

## 2. Carbohydrate

Carbohydrate is the most abundant macronutrient in the human diet, providing 45–70% of daily calories [3,4]. However, carbohydrates are not considered an essential nutrient for humans [17], and their increased consumption has recently been associated with “carbotoxicity” [6,7]. Several human trials have shown that reducing carbohydrate consumption is beneficial for metabolic health, and ketogenic diets that severely limit carbohydrate intake to <10% daily energy are effective in producing weight loss and improving the glycaemic profile in type 2 diabetes [18]. Furthermore, a recent large-scale epidemiological study (Prospective Urban Rural Epidemiology (PURE) study) showed that a high carbohydrate intake (highest (quintile 5) vs. lowest quintile (quintile 1)) is associated with increased risk of mortality and dyslipidaemia [7,19]. Together, these observations make a case for revising current dietary guidelines to reduce the total amount of carbohydrate consumption and decrease the proportion of daily energy derived from carbohydrate.

### 2.1. Types of Carbohydrates and Their Metabolic Effects

In addition to total carbohydrate intake, evidence suggests that the “type” of carbohydrate eaten is also an important determinant of metabolic outcomes [20,21,22,23,24]. Fibre, starch, sucrose, and high fructose corn syrup (HFCS) are the major types of carbohydrates in the diet of adult humans [20,21,22,23,24]. Fibre is not a major source of daily energy [4,25]. It is composed of polysaccharides derived from plant cell wall, whole grains, fruits, and vegetables, and includes resistant starch, inulin, oligofructose, polydextrose, and galactooligosaccharides [21]. Studies have shown that consuming fibre-rich foods leads to metabolic improvements such as weight loss and improved insulin sensitivity, and these changes are associated with an increased abundance of beneficial bacteria in the gut microbiome [21,22,26]. Currently, the daily intake of fibre for the U.S. population is 16 g/d; for European adults, the average intake is 20–25 g/d, which is less than the recommended fibre intake by the U.S. Department of Agriculture (USDA) of 25–38 g/d [25,27].

In terms of energy, starch, sucrose, and HFCS account for most of the carbohydrate-derived calories in adult Western diets [4]. Starch is a polysaccharide of glucose that exists as either linear chains of glucose monomers joined to each other by α-1,4 glycosidic bonds (amylose) or in branched form containing both α-1,6 and α-1,4 linkages (amylopectin) [28]. Sucrose (table sugar) is a disaccharide of glucose and fructose, while HFCS is a mixture of glucose and fructose in monosaccharide form [4,29]. HFCS is produced by treating corn syrup with the enzyme “glucose isomerase” that converts glucose derived from corn starch to fructose [24]. In the United States, successful glucose isomerisation on an industrial scale in 1967 led to the replacement of sucrose by HFCS in processed foods because of greater availability and lower prices of corn [24,30]. While sucrose is still the predominant caloric sweetener in most parts of the world, HFCS accounts for ~40% of caloric sweeteners added to foods (e.g., canned fruits, jellies, and baked goods etc) and drinks in the United States [24,31,32]. The most commonly used forms of HFCS are HFCS-55 (containing 55% fructose and 45% glucose) and HFCS-42 [31,32].

Glucose and fructose are the monomeric building blocks of the major energy-providing dietary carbohydrates [4]. Glucose is a source of energy for all tissues, but fructose is not essential for human metabolism and it is rarely consumed in isolation [23,31]. Fructose can be synthesised endogenously in the liver from glucose via the polyol pathway [33]. In general, most Western diets contain over three-fold more glucose than fructose [34]. In contrast to fructose found in nutrient-rich whole fruits, consuming fructose-derived calories from nutrient-poor sources such as caloric sweeteners (sucrose, HFCS and pure fructose) added to processed foods and beverages have been associated with adverse metabolic consequences [23,24]. For example, epidemiological data from the United States suggest that increased consumption of fructose, especially from HFCS, is responsible for the rapid rise in obesity prevalence over the last 40–50 years [24,35]. The increase in fructose and HFCS consumption closely paralleled the rise in prevalence of overweight and obesity in the United States between 1960 and 2000 [24,35]. In addition to obesity, higher intakes of sucrose, HFCS, and fructose, especially from beverages, increase the risk of developing type 2 diabetes [36,37,38,39]. Similarly, experimental studies in animals and humans have shown that consuming extra calories from fructose-containing sweeteners promote obesity, dyslipidaemia, fatty liver, and insulin resistance [23,40,41,42,43,44,45,46]. These adverse effects are more obvious when fructose is derived from beverages such as soda drinks, sweetened milk drinks, fruit juices, and iced tea [45,47,48,49]. This is because, compared with solid foods, the biological regulation of total energy intake is less precise when sugars are consumed as fluids [4,50,51]. Furthermore, fructose intake stimulates parts of the brain associated with feelings of reward and pleasure, and these hedonic effects of fructose can promote increased energy consumption [52]. Functional MRI scanning in healthy volunteers after a glucose or fructose drink showed that fructose stimulated greater reactivity to food cues in the visual cortex and left orbital frontal cortex [53]. Behavioural studies in animals have also shown signs of dependence, such as bingeing, withdrawal, craving, and cross-sensitisation to other drugs with intermittent sucrose administration [54]. However, contrary to the effects of consuming excess calories from fructose, results of the studies comparing the metabolic consequences of fructose-containing caloric sweeteners with other types of carbohydrates (e.g., glucose and starch) have been inconsistent and contradictory [43,45,55,56]. This has led to strong controversy in sugar research and has caused debate as to whether fructose per se is harmful for health beyond its contribution to excess calories [45,55].

### 2.2. Glycaemic Index and Metabolic Effects of Carbohydrates

In addition to molecular structure, glycaemic index (GI) is also used as a marker of carbohydrate quality. GI is a measure of the glycaemic response to consuming a food item containing 50 g of carbohydrate. It is measured as the incremental AUC (iAUC) for blood glucose over a two-hour timeframe and expressed as a percentage of the iAUC of a reference food, usually pure glucose solution or white bread, which are assigned GI values of 100 (GI = (iAUC_food item_/iAUC_reference_) × 100) [57,58]. A related parameter is the glycaemic load (GL), which is the product of GI and available carbohydrate in a given amount of food (GL = GI × available carbohydrate in the food item) [57]. Foods with GI values of ≤55 are classified as low GI foods; those with GI of 56–69 are medium; and those with GI ≥ 70 are labelled as high GI foods [57]. Most starchy foods have a GI of >70, and Thai Jasmine rice has a GI of 100 [59]. Compared with low GI diets, consuming high GI diets would produce a greater spike in postprandial blood glucose and insulin concentrations [58]. This will result in the rapid utilisation of glucose by peripheral tissues, leading to a faster return of feelings of hunger, and the spike of insulin could lead to greater anabolic effects of insulin such as lipogenesis and increased lipidaemia. This could lead to adverse consequences such as obesity and insulin resistance in the long-term [58]. In addition, emerging evidence suggests that high GI diets could have transgenerational effects due to epigenetic changes induced in the placental and foetal tissues [60,61].

Results of experimental studies comparing the effects of low vs. high GI diets on subjective measures of satiety, fullness and appetite have been inconsistent [57]. Although some studies have reported higher ratings of fullness in subjects consuming low GI diets [62,63], others have reported no significant differences in satiety on low vs. high GI foods [64,65,66]. Furthermore, data from studies investigating the relationship between dietary GI and obesity have been equivocal [57]. A cross sectional study in young Japanese women showed a positive correlation between GI, GL, and body mass index (BMI) [67]. Similarly, in a study involving 175 subjects with type 2 diabetes, there was a positive association between dietary GI and waist circumference [68]. In an eight-week weight loss trial including 30% energy restriction, subjects on a low GI diet lost significantly more weight than those in the high GI group [69]. At a molecular level, a study in male subjects given a high or an isocaloric low GI meal after exercise, showed that glucose and insulin AUC were increased, while gene expression of fatty acid transporter CD36 in skeletal muscle tissue was significantly reduced after consuming a high GI meal [70]. This indicates reduced fat metabolism after consuming a high GI meal [70]. Contrary to these observations, no association between dietary GI and BMI was observed in a study of Spanish adults [71]. Moreover, in older adults from the United Kingdom, no association was reported between GI and body weight or BMI [72]. This was similar to the data for elderly subjects from rural Spain, showing no association between GI or GL and waist circumference or BMI [73]. The results of these epidemiological studies are consistent with several weight loss trials, including those that involved energy-matched interventions, which reported no differences in weight loss on subjects maintained on high vs. low GI diets [74,75,76,77]. The evidence from the prospective studies for the association between dietary GI and risk of developing type 2 diabetes [78,79,80,81], and results of experimental trials exploring the link between GI and markers of glycaemic control, have also yielded contradictory evidence [75,82,83].

Possible reasons for the inconsistent evidence about the metabolic outcomes associated with high vs. low GI diets include confounding factors such as the higher fibre content of low GI diets and the use of food frequency questionnaires in observational studies for collecting self-reported dietary data [57]. Another issue with the concept of GI is that foods containing sucrose or HFCS can have low GI values (e.g., the GI of pure fructose is 19) but still be adverse for metabolic health [22,84]. However, a recent meta-analysis of prospective cohort studies involving healthy adults showed a 90% increase in the risk of type 2 diabetes when comparing the lowest to the highest GI exposure across the globe (GI of 48 vs. 76) [85]. The strong association with high dietary GI and risk of type 2 diabetes was independent of levels of dietary fibre intake [85]. Furthermore, a recent publication from the high-profile PURE study involving data for 137,851 subjects from 20 countries on five continents showed an increased risk of cardiovascular disease and death with high GI of the diet [86]. When comparing the highest vs. lowest quantiles of GI, the risk of a composite outcome of major cardiovascular event and death was increased both in subjects with (hazard ratio 1.51) and without (hazard ratio 1.21) pre-existing cardiovascular disease [86]. The WHO commissioned a systematic review and meta-analysis of prospective studies and randomised clinical trials to investigate the association between the intake of dietary fibre, whole grains, GI, and cardiometabolic disease [22]. When compared with people that consumed low fibre diets, coronary artery disease, type 2 diabetes and all-cause mortality were decreased by 15–30% in high fibre consumers [22]. The observational data for mortality translated into 13 fewer deaths for highest vs. lowest fibre intake per 1000 participants over the course of the studies [22]. In experimental trials, high fibre intake resulted in benefits such as lower body weight and lower levels of cholesterol [22]. The results of whole grain consumption were similar to fibre intake, but a reduction in the risk of type 2 diabetes with low vs. high GI diet was modest when compared with data for fibre intake [22]. Similarly, data from clinical trials showed inconsistent effects of GI index on cardiometabolic outcomes [22]. Thus, overall, the evidence for using low GI diets as a strategy for the prevention and treatment of cardiometabolic disease is not as strong as that for high fibre intake [22,57]. Further well-controlled studies are required to formulate dietary guidelines in the light of the impact of GI on cardiometabolic outcomes.

### 2.3. Molecular Mechanisms of Metabolic Benefits of Fibre Intake

The mechanisms of beneficial effects of dietary fibre on gut microbiota composition and function as well as on host metabolism are well established [21]. Bacteria in the cecum and colon have the enzymatic machinery to ferment dietary fibre into short-chain fatty acids (SCFAs), mainly acetate, butyrate, and propionate at a ratio 3:1:1 which are absorbed into systemic circulation. Binding of these fatty acids to G-protein-coupled receptors (GPCRs) in various tissues is thought to mediate the metabolic effects of fibre intake [21,25,87,88]. Butyrate, acetate, and propionate bind, with the highest selectivity, to the G-protein coupled receptors GPR109A, GPR43 and GPR41, respectively [87,88,89]. Butyrate is the major source of energy for enterocytes in the gut, while propionate and acetate might be metabolised in the liver. Acetate has the most marked systemic effects with plasma concentrations reaching 19–160 µM vs. 1–13 µM for butyrate and propionate [21].

Resistant starch, made of linear amylose chains in granules that are resistant to digestion by intestinal enzymes, is one of the most commonly used types of fibre in research. Mice fed on resistant starch had increased glycolysis and fatty acid oxidation in liver [90]. It is also known to beneficially reshape their gut microbiota by increasing the abundance of Bacteroides, Akkermansia, and Bifidobacterium while reducing Firmicutes [90]. Similarly, in the setting of isocaloric diets, replacing 30% energy from starch with resistant starch for 12 weeks produced an increase in the concentration of all three SCFAs in cecum, reduced body weight and adiposity by increasing energy expenditure and oxidative lipid metabolism, and increased insulin sensitivity in mice [21]. Compared with native starch-fed mice, mice consuming a high resistant starch diet had drastically different plasma metabolome, including a 22-fold increase in the circulating concentrations of the tryptophan-derived metabolite indole propionate [91]. Treatment of rats with indole propionate resulted in an improved glycaemic profile [92]. In humans, the consumption of resistant starch lowered cholesterol and body fat, substantially increased acetate and propionate concentrations in plasma, and improved insulin sensitivity in subjects with metabolic syndrome [89,93].

Intake of SCFAs reproduces most of the benefits associated with increased fibre intake. Most of the mouse studies have focused on the high fat diet (HFD) model of obesity, and diets containing 5% (wt./wt.) of individual SCFAs have been commonly used. Supplementation of HFD diets with SCFAs either completely or partially prevented HFD-induced obesity without any changes in physical activity [94]. Acetate was found to be most effective of all the SCFAs in reducing body weight in some studies, while others showed an equal effect for all three SCFAs [94]. In addition, a 12 week HFD mouse study showed an increased energy expenditure and fatty acid oxidation secondary to increased AMPK activity and UCP2 expression in the liver with SCFAs supplementation [95]. This led to about a 1.5-fold higher glucose infusion rate in animals fed any of the three SCFA-containing diets during hyperinsulinemic clamp studies, indicating improved insulin sensitivity [95]. Improvement in glucose tolerance, lower fasting insulin and blood glucose, mitochondrial biogenesis, beige adipogenesis, and increased UCP-1 expression in brown fat have also been reported with SCFA intake [96,97,98]. Similar to resistant starch consumption, a decrease in the proportion of Firmicutes and an increase in Bacteriodes was observed with SCFA intake [94]. In obese humans, consumption of 1.5 g/day acetate for 12 weeks reduced body weight and BMI and caused a 3.5% decrease in abdominal fat area [99].

GPCR activity is required for metabolic benefits of SCFAs. HFD feeding reduces the expression of GPR-41, 43 and 109A in the adipose tissue of animals [97,100]. Studies using knockout mice have been critical to understanding the mechanisms of the metabolic effects of SCFAs. GPR41^−/−^ mice were found to have a lower resting heart rate and UCP1 expression in brown fat, and their energy expenditure remained unchanged after propionate treatment [87]. Mice lacking GPR109A were obese and developed hepatic steatosis on a chow diet [101]. GPR43^−/−^ mice were found to be obese on a chow diet and had higher body weights than wild-type mice on an HFD [88]. This translated into impaired glucose tolerance and reduced sensitivity to exogenous insulin in clamp studies [88]. In contrast, overexpression of GPR43 in adipose tissue protected from HFD-induced obesity, lowered fasting blood glucose, increased energy expenditure and fat metabolism, and reduced liver triglycerides [88]. Treatment with antibiotics or housing animals in germ-free conditions completely blocked the effects of GPR43 deletion and overexpression, suggesting that these effects are microbiome-dependent [88].

### 2.4. Molecular Mechanisms of Adverse Effects of Carbohydrate Intake

Diverse mechanisms have been proposed to mediate the molecular effects of carbohydrate intake. Rapid digestion of simple carbohydrates produces spikes of insulin secretion that cause a dip in blood glucose levels and stimulation of appetite [6]. Moreover, carbohydrate-induced insulin release may facilitate fat deposition by stimulating lipogenesis and inhibiting lipolysis [3]. The ketone or aldehyde moiety of carbohydrate molecules can react with the amino group of lysine in proteins or DNA bases, or with a free hydroxyl group of lipids to generate reactive oxygen species (ROS) [43]. Increased production of ROS has been linked to insulin resistance and pancreatic beta-cell dysfunction in diabetes [102]. Dihydroxyacetone phosphate and methylglyoxal produced from cellular glucose metabolism can also react with free amino groups found in proteins to form advanced glycation end products (AGEs) [6,103]. AGEs are widely reported to mediate complications of diabetes in several tissues [6].

**Figure 1:**
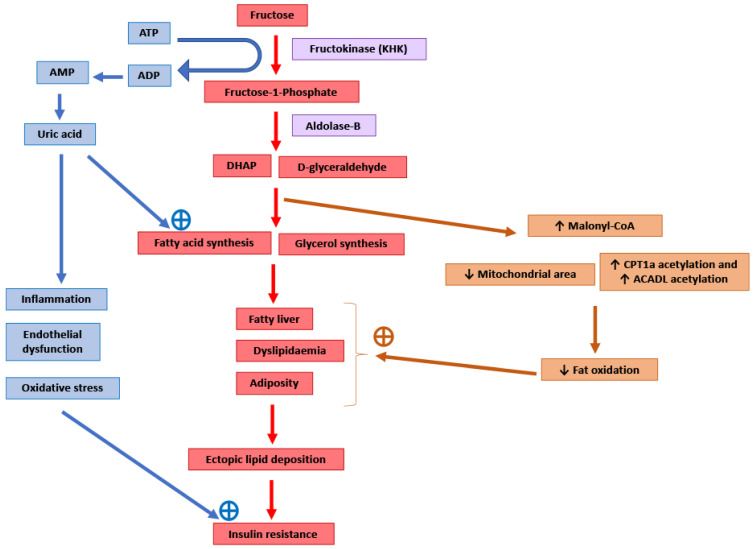
Potential molecular mechanisms mediating the adverse consequences of high fructose intake. DHAP: Dihydroxyacetone phosphate. CPT1a: Carnitine palmitoyltransferase 1 (hepatic isoform a). ACADL: Long-chain specific acyl-CoA dehydrogenase.

It has been suggested that the pro-lipogenic nature of fructose metabolism in the liver makes it more detrimental for metabolic health than other carbohydrates [6,43] (Figure 1). Around 50–75% of the fructose absorbed by the intestines is metabolised in the liver [104]. After entering the hepatocytes through GLUT2 and GLUT5 transporters, the enzyme ketohexokinase (KHK; also called fructokinase) phosphorylates fructose to fructose-1-phosphate [6,105]. The enzyme aldolase-B then converts fructose-1-phosphate into D-glyceraldehyde and dihydroxyacetone phosphate [6,105]. Further downstream metabolism of these three-carbon metabolites can lead to fatty acid synthesis via acetyl-CoA or generate a glycerol backbone of triglycerides [105]. Contrary to glucose, fructose metabolism in the liver is not tightly regulated by insulin signalling or by negative feedback from ATP and citrate, and this absence of feedback signals facilitates the potent induction of de novo lipogenesis (DNL) [6]. Comparison of DNL induction by high glucose vs. fructose intake for six days in humans showed a fractional DNL rate of 2% with glucose and up to 10% with fructose [43,106]. Studies in mice where a high fat diet was supplemented with either a 30% fructose or glucose solution showed that both these monosaccharides activated the lipogenic factor ChREBP in the liver [107]. However, fructose additionally activated the lipogenic transcription factor SREBP1c and genes associated with fatty acid synthesis [107]. Glucose activates ChREBP, which leads to increased glycolysis and fatty acid synthesis [108]. Activation of the glucose-response activation conserved element (GRACE) domain of ChREBP by glucose metabolites leads to the binding of ChREBP to carbohydrate response element (ChoRE) sequences present on the promoter DNL pathway genes such as *Fas*, *Acc* and *Scd1* and increases their mRNA expression [109,110]. Fructose-induced hyperinsulinaemia and the resultant increase in insulin signalling increases the expression of DNL genes by activating SREBP-1c in the liver [111]. Mice with defects in the processing of SREBP-1c in ER and Golgi (required for SREBP-1c activation and its nuclear translocation) had markedly reduced insulin-induced DNL [112,113]. For example, deficiency of Scap, a protein that escorts SREBPs from ER to Golgi, reduced liver fat and triglyceridaemia in high fat diet and high sucrose diet models of rodent obesity [114]. Fructose-induced DNL results in the generation and secretion of very low-density lipoprotein (VLDL) particles into the systemic circulation that contributes to hypertriglyceridaemia and adversely affects lipid profile [45]. In addition, compared with glucose solution, the supplementation of a high fat diet with fructose solution reduced fatty acid oxidation in mice [115]. This was thought to be mediated by a fructose-induced increase in concentrations of hepatic malonyl-CoA (an inhibitor of fat oxidation), mitochondrial dysfunction characterised by reduced mitochondrial area, and increased inhibitory acetylation of fat oxidation pathway factors CPT1a and ACADL [115]. Overall, these changes in fat metabolism culminate in hepatic steatosis, increased visceral adiposity, and ectopic lipid deposition in liver and muscle tissue [42,44,45]. The toxic lipid species (ceramide and diacyl glycerol) generated secondary to ectopic lipid accumulation are proposed to eventually inhibit insulin signalling, leading to insulin resistance [45].

The enzyme KHK is the major mediator of metabolic outcomes of high fructose intake, and recent research has identified this enzyme as a potential therapeutic target for the management of obesity and fatty liver [104,115,116]. KHK exists in two isoforms: KHK-C and KHK-A. The highly active isoform “C” is expressed mainly in the liver, kidney and intestines, and metabolises the majority of the fructose absorbed from the diet [104,116]. The isoform “A” has widespread but low levels of tissue expression and low affinity for fructose [104,116]. Mice provided with a 30% fructose solution to drink for 10 weeks as well as obese human subjects with non-alcoholic steatohepatitis (NASH) showed increased hepatic expression of KHK and downstream lipogenic genes *Acaca, Acly* and *Scd1* [107]. Knockdown of hepatic KHK by siRNA or combined deletion of KHK-A and KHK-C protected the mice from high fructose-induced obesity, fatty liver, glucose intolerance and insulin resistance [104,107,116,117]. The activity of KHK results in the breakdown of ATP to ADP and AMP, and this ATP depletion may result in an increase in phosphofructokinase (PFK) activity, leading to the increased utilisation of glucose for glycolysis and downstream DNL [118]. ATP degradation also activates AMP deaminase which converts AMP to inosine monophosphate, which is further converted into hypoxanthine, xanthine, and eventually into uric acid [6]. Uric acid is a proinflammatory metabolite that induces mitochondrial oxidative stress and inhibits aconitase in the citric acid cycle [6,119]. This stimulates DNL by the accumulation of citrate and the stimulation of ACLY and FASN enzymes [119]. Serum uric acid levels correlated positively in non-diabetic human subjects with the severity of hepatic steatosis [119]. Allopurinol, a xanthine oxidase inhibitor that blocks the conversion of xanthine to uric acid, reduced hepatic steatosis in a mouse model of metabolic syndrome [119]. Additionally, uric acid causes endothelial dysfunction that could lead to hypertension and insulin resistance [48].

Sucrose (or fructose) are often added to high fat rodent diets to model Western diets that are rich in fat and sugar [116]. This is because adding sucrose to a high fat diet makes the metabolic impairment more severe [120,121]. In the liver, in addition to steatosis, this combination of sucrose and fat facilitates the induction of mild inflammation and fibrosis [116,122]. This indicates that fructose interacts with other nutrients in the diet to influence the metabolic phenotype. However, the mechanistic underpinnings of the interaction of fructose (and glucose) with fat and protein have not been examined in detail.

## 3. Fat

Dietary fats have been linked to the development of a number of clinical metabolic disorders such as obesity, insulin resistance, type 2 diabetes and cardiovascular disease. Deposition of triglycerides within tissues other than adipose tissue has long been proposed as an important indicator of these medical problems [123]. Fatty acids, although often seen as detrimental, are critical for life, having extremely important functions in membrane structure and function, cell signalling, steroid hormone production, as well as in metabolism and energy production.

### 3.1. Types of Fatty Acids and Their Metabolic Effect

Free fatty acids (FFAs) are hydrophobic molecules and are usually grouped according to the length and saturation of their side chain; these being short- (SCFA, 2–6 carbons), medium- (MCFA, 8–12 carbons), long- (LCFA, 14–18 carbons) and very long- (VLCFA, 20–26 carbons) chain fatty acids. High dietary and plasma levels of LCFA have been associated with obesity and insulin resistance, while MCFAs are not strongly associated with deterioration in metabolic health [124]. Within these groups, FAs can be either saturated (no carbon double bonds), monounsaturated (one double bond) or polyunsaturated (more than one double bond). The most common forms of FAs circulating in human plasma are palmitic acid (C16:0), palmitoleic (C16:1), stearic acid (C18:0), oleic acid (C18:1) and linoleic acid (C18:2) [125,126].

Fats are often termed “good” or “bad” fats due to their reported effects on health. Studies have shown that saturated FAs (SFAs) are associated with poorer metabolic and cardiovascular outcomes, while polyunsaturated fats (including omega-3) are associated with better outcomes [8,9]. Thus, of the recommended 20–35% of calories [5] coming from fats, the predominant form should be from the “good” column, such as foods containing monounsaturated fats, e.g., avocados, nuts, olive oil, chia seeds, and fatty fish, which are sources of polyunsaturated fats. Limiting the amount of the “bad” saturated fat (dairy, animal fats), and especially foods containing *trans*-FA (hydrogenised margarines and oils), is also recommended for metabolic health [127].

Studies investigating high fat diets in animal models show that these diets can cause weight gain, insulin resistance and metabolic disease [128]. Diets that are often used to induce these metabolic changes contain 60% fat, or a 45% fat diet which often contains a high amount of sucrose. These obesogenic diets often use lard as the major source of fat, which contains high levels of the detrimental long-chain fatty acids palmitic and stearic acids [129]. As previously mentioned, these diets cause the deposition of fat not only in adipose tissue, but also in non-adipose tissue, such as the liver and muscle. This “ectopic” deposition of triglycerides has been associated with decreased insulin-stimulated glucose uptake into muscle and adipose tissue [130], while the liver continues to release glucose into the circulation due to the failure of increased insulin levels to “switch off” gluconeogenesis and limit hepatic glucose output [130]. All these effects contribute to the development of fasting hyperglycaemia. In human studies, acute infusions of fatty acids have also been found to produce insulin resistance in muscle and liver [131,132].

As mentioned above, not all fatty acids are associated with detrimental outcomes. Rodents fed diets containing mostly medium-chain fatty acids (MCFA) have not been associated with metabolic alterations or insulin resistance, even when eaten in excess [133,134,135]. That is, animals had less adiposity and better glucose tolerance [134,135], and while the liver had an intermediate level of insulin sensitivity, MCFA-fed animals had preserved muscle and adipose tissue insulin sensitivity compared to animals fed an LCFA diet [135]. Animals fed diets with a fat source from fish oil also did not produce the same detrimental effect as dietary LCFAs, having reduced fat mass, better glucose tolerance, and lower triglyceride levels in the liver [129,136,137]. Thus, in addition to total dietary fat content, fat source is an important factor in determining metabolic outcomes of diets with high fat content.

Another interesting aspect of high fat diets to note is the growing trend of using ketogenic diets for weight loss. Ketogenic diets traditionally are very high in fat (often 85%) with very low levels of carbohydrates (<10%), and were originally designed for the treatment of childhood epilepsy [138]. A side effect of this diet was weight loss, which is likely due, in part, to the appetite-suppressing effect (and thus calorie restriction in an ad libitum setting) of ketone bodies [139]. In another study that investigated the use of eucaloric high fat diets (i.e., diets aimed at maintaining body weight), no alterations were seen in insulin sensitivity in either humans and mice [140]. Thus, more research is needed, but it seems that overconsumption of saturated, long chain, fats in a hypercaloric setting is likely responsible for the observed insulin resistance.

### 3.2. Molecular Mechanisms Involved in HFD-Induced Insulin Resistance

**Figure 2:**
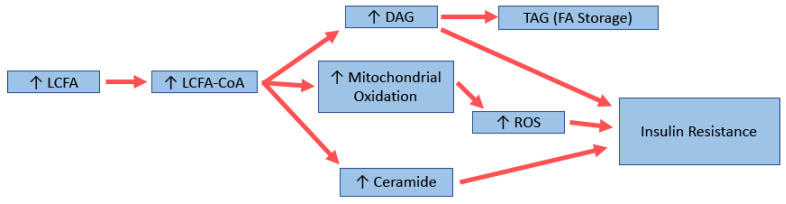
Potential molecular mechanisms for long-chain fatty acid (LCFA)-induced insulin resistance. DAG: diacylglycerol; TAG: triglyceride; ROS: reactive oxygen species.

Triglycerides are a biologically inert form of storing FA, and thus are unlikely to be the driver of insulin resistance that occurs after high fat feeding. Evidence shows that it is more likely that the bioactive lipid intermediates, including diacylglycerides (DAGs) and ceramides, play a more causal role (Figure 2). Other factors, including excess reactive oxygen species (ROS) generation from increased mitochondrial oxidation leading to oxidative stress, may also play a role. Elevated levels of DAGs have been found in liver, muscle, and adipose tissue in insulin-resistant states [130,141]. It is thought that elevated DAG levels can increase the activity of various protein kinase C (PKC) isoforms, which then interfere with the insulin signalling pathway. In muscle, for example, evidence suggests that PKCθ phosphorylates inhibitory serine residues on IRS proteins which leads to decreased signalling and reduced glucose uptake [141]. In the liver, PKCε is thought to be the major isoform involved [142]; however, this has recently been challenged [143,144]. Whatever the mechanism, there is a strong link between increased DAGs and the insulin-resistant state.

Ceramides have also been shown to be increased in the muscle and liver of insulin-resistant animals and humans [130,145]. A recent review by Summers (2020) describes the many mechanisms in which elevated levels of ceramide can cause/contribute to the development of insulin resistance, including through increased fatty acid uptake and production, decreased glucose uptake and lipolysis, and alterations in mitochondrial fission and efficiency [146]. Manipulating ceramide levels has been shown to have positive effects, with one study showing decreasing skeletal muscle levels of 18:0 ceramide by genetic manipulation was associated with beneficial effects in mice [147], and lower levels of ceramide 18:1/18:0 were found in a distinct population of metabolically healthy (and thus insulin-sensitive) obese people [148]. Interestingly, in the liver, increases in very long chain ceramides have been associated with better glucose tolerance in different strains of mice [149]. In another study, lipidomic analysis of muscle from mice fed MCFA has shown elevated levels of 18:0 ceramides, which is usually associated with insulin resistance, but increased levels of 24:0 ceramide, associated with better outcomes [150]. Thus, although there is strong evidence for ceramides to be involved in insulin resistance, further research is needed to elucidate the mechanisms for each species and its impact on insulin-resistant states.

With an increase in fat intake, there is an increase in mitochondrial metabolism. It has been shown that HFDs can cause increases in the mitochondrial machinery and oxidative capacity in rodents [130]. However, this also increases the generation of the reactive oxygen species including superoxides, which in muscle is produced primarily by electron leakage from complex 1 and complex 3 of the mitochondrial electron transport chain [151,152]. Decreasing oxidative stress through various mechanisms, e.g., by overexpressing manganese superoxide dismutase (SOD2; a superoxide scavenger [153,154]), or by treatment with antioxidants [154], can lead to improved insulin sensitivity. Interestingly, while signs of oxidative stress were noticed in muscles of mice fed LCFA diets, including increased glutathione peroxidase activity, protein carbonylation, and lipid peroxidation, animals fed MCFAs had levels similar to that of controls [134]. Diets supplemented with omega-3 FA have also shown a decrease in liver oxidative stress when compared to HFD [155]. The ability of the mitochondria to adapt fuel oxidation to fuel availability is termed metabolic flexibility [156,157]. In obesity, this ability is usually impaired [157]; however, it is retained in metabolically healthy but obese people [158]. Interestingly, a recent study showed that low metabolic flexibility predicts future weight gain in normal-weight individuals [159]. An important regulator of this glucose/fatty acid switch is pyruvate dehydrogenase (PDH), which itself is regulated in part by pyruvate dehydrogenase kinase 4 (PDK4) [160]. PDK4 has been found to be upregulated in rodents fed a high fat diet high in SFA [161,162,163], as well as in insulin-resistant humans [164,165]. Although this seemed a good target for the treatment of insulin resistance, Small et al. (2018) showed that acute treatment of high fat-fed rats with dichloroacetate (DCA), an inhibitor of the PDKs, had little effect on insulin-stimulated glucose uptake, although it did increase glucose oxidation [166].

Thus, although a lot of work has been done in the area of dietary fat and insulin resistance, several key questions regarding the precise mechanisms mediating divergent effects of different types of FAs on metabolic signalling remain unanswered.

## 4. Protein

When looking at potential nutritional interventions for lifestyle diseases such as insulin resistance and obesity, a greater emphasis is typically placed on studying the changing patterns of fat and carbohydrate consumption. Until recently, the role of dietary protein in the emergence of these diseases has been largely overlooked for two major reasons [167]. Firstly, protein contributes a much smaller component of an individual’s overall dietary energy budget in comparison to non-protein counterparts. Secondly, protein intake has remained far more stable over time when compared to fat or carbohydrate [168]. Rather than indicating that protein plays little to no role in appetite regulation and energy balance, it is this long-standing stability of protein intake that provides an insight into highly conserved mechanisms that drive feeding behaviour. Evidence shows that when there is a nutritional imbalance observed in the levels of protein, carbohydrate and fat within diets, animals tend to prioritise intake of protein more strongly than non-protein energy. This phenomenon has been termed as “protein leverage” [169], and has been observed in a wide range of species, from insects to humans [170,171,172,173]. Through protein leverage, excess energy is consumed in diets with a lower ratio of protein to non-protein macronutrients, while an energy deficit is incurred (in ad libitum conditions) when protein content is high, in order to defend the protein target. The absolute intake of protein remains relatively constant, while the intake of fat and carbohydrate varies substantially as a means to compensate in both cases [169].

### 4.1. Protein, Branch-Chained Amino Acids, and Their Metabolic Effects

Due to the hyperphagic feeding behaviour that humans and other animals display in protein-dilute situations, one of the clear consequences of protein leverage is obesity. It has long been recognised that obesity is a major risk factor in the development of dyslipidaemia and associated chronic metabolic disorders such as insulin resistance and diabetes, particularly in cases where energy expenditure is not increased [174]. Through methods such as dietary restriction, exercise, and fasting [175,176,177], the first line of defence used against combating obesity is often weight loss, because it has been shown to improve outcomes such as insulin sensitivity, among other related co-morbidities [178,179,180]. To achieve weight loss, it is often recommended that overweight and obese individuals increase their protein intake in order to promote lean, fat-free mass and decrease overall energy intake [181]. Some studies have shown that a short-term period on a high protein diet can improve insulin sensitivity in subjects with obesity and insulin resistance [182,183,184]. While these high protein diets have been associated with weight loss, as a long-term dietary intervention it is important to consider that there is growing evidence that a prolonged exposure to high protein diets places individuals at a higher risk of developing insulin resistance, type-2 diabetes and increased mortality in rodents and humans [14,185,186]. Conversely, chronic consumption of low protein diets is associated with better metabolic health and increased survival [13,187,188].

It is clear that dietary protein plays a key role in mediating metabolic health. Recent work, however, has highlighted that within proteins, the quantities and mixtures of amino acids are themselves powerful modulators of metabolism [10,11,12,13,14,15]. Of the 21 proteinogenic amino acids, nine are “essential” and must be supplied by diet; six are “conditionally essential”; and another six are “non-essential” [189,190]. These amino acids are also classified according to their biochemical structure (e.g., sulphur-containing or branched chain amino acids) [189,190]. Restriction of specific amino acids, such as the sulphur-containing amino acids, methionine and cysteine, have been shown to improve metabolic health and lifespan in mice and rats through glutathione-mediated resistance to oxidative stress [191,192]. Dietary threonine restriction has also been shown to improve glycaemic control and reduce hypertriglyceridaemia in mice through the induction of FGF21 [11]. However, of the essential amino acids, particular attention has been given to the dietary manipulation and circulating blood levels of the branched chain amino acids (BCAAs) isoleucine, leucine, and valine, due to their central role in protein synthesis and influencing key signalling pathways such as insulin, mTOR, and FGF21 [193].

As essential amino acids, BCAAs cannot be endogenously synthesised and must be acquired from the diet. Once ingested, their fates are to become (i) direct substrates for protein synthesis; (ii) signalling molecules that stimulate anabolic pathways; and/or (iii) catabolised for ATP generation in an energy-limited environment [194]. Unlike other amino acids, their initial metabolism is in skeletal muscle rather than the liver, where they play a central role in stimulating anabolic pathways in muscle [195]. These unique characteristics make BCAAs an important signal reflecting the balance between dietary protein intake and endogenous protein catabolism [196]. Unlike carbohydrates and fats, excess protein/amino acids cannot be directly stored. Instead, BCAAs are catabolised, and the metabolites generated are used for ATP production or storage as triglycerides or glycogen [195,196]. Together, the circulating levels of BCAAs and their related metabolites, such as the branched-chain α-ketoacids and acylcarnitines, have been proposed as markers of metabolic dysfunction [197].

In both humans and rodents, elevated circulating levels of BCAAs and related metabolites are positively associated with cardiometabolic risk factors such as insulin resistance, elevated blood glucose, obesity, and dyslipidaemia [13,14,194,197,198,199,200,201,202]. However, it remains unclear if the relationship between BCAAs and cardiometabolic risk factors are a reflection of dietary factors (e.g., excess protein intake), or if elevated circulating BCAAs are a cause or consequence of dysfunctional glucose, insulin, or lipid metabolism. Indeed, some studies have shown that elevated BCAA levels, whether in the post-prandial or fasting state, closely reflect dietary BCAA intake [10,14,203], and that BCAAs alone were not the strongest metabolite signature associated with insulin resistance in different strains of mice [204].

### 4.2. Molecular Mechanisms of Protein/BCAA Induced Insulin Resistance

**Figure 3:**
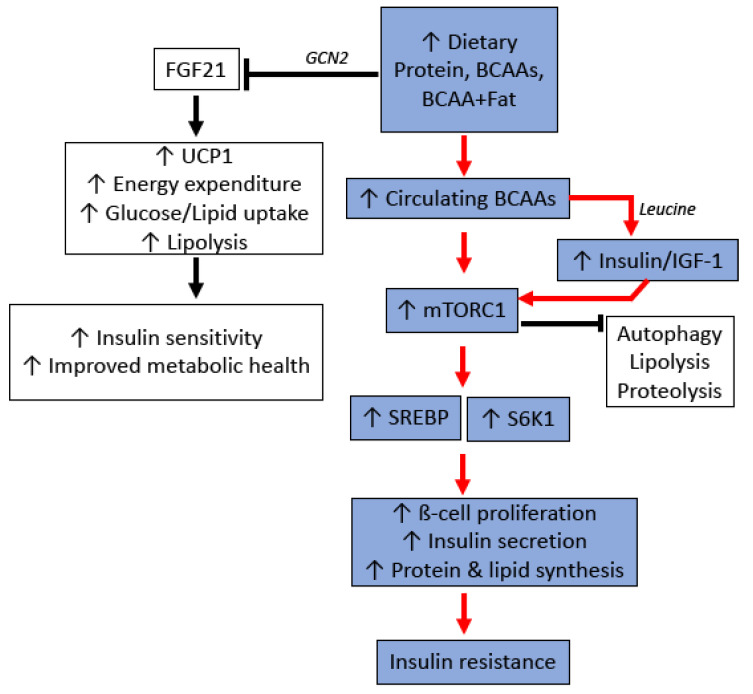
Potential molecular mechanisms for protein and branched chain amino acid (BCAA)-induced insulin resistance. GCN2: General control nonderepressible 2 kinase; FGF21: Fibroblast growth factor 21; UCP1: Uncoupling Protein 1; IGF-1: Insulin-like growth factor-1; mTORC1: Mammalian target of rapamycin complex 1; SREBP: Sterol regulatory element-binding protein; S6K1: Ribosomal S6 Kinase 1.

Although disentangling this complex relationship between diet, circulating BCAA levels and cardiometabolic health remains to be resolved, it is clear that a major mechanism linking protein and BCAAs to metabolic dysfunction is the activation of mTOR (Figure 3). The mTOR complex exists in two major forms: mTORC1, which integrates nutritional signals from the environment to control key cellular processes such as protein synthesis and autophagy; and mTORC2, regulating hormonal signals such as insulin and IGF-1 [205]. The relationship between mTOR and insulin sensitivity, however, integrates both these complexes, with hormonal feedback from the insulin/IGF-1 pathway necessary for the mTORC1 activation. Reducing total protein intake or BCAAs has been shown to reduce mTORC1 activation in the liver, muscle, and white adipose tissue [185,206], and it is generally accepted that chronic hepatic mTORC1 signalling contributes to insulin resistance via the inhibition of insulin receptor substrate-1 (IRS-1) [207]. Interestingly, BCAA-mediated mTOR activation and insulin resistance appears to be influenced by the background nutritional composition of the diet. mTOR activation and insulin resistance in rats is increased with BCAA supplementation, but only when combined with a diet that was also high in fat [197,198]. When given a high carbohydrate, low fat diet, BCAA supplementation did not appear to increase hepatic mTOR activation, or influence BCAA metabolism or related metabolites [10]. Additionally, it is notable that long term exposure to low protein diets promotes the secretion of FGF-21, an endocrine signal which is now recognised to improve glucose metabolism and reduce insulin resistance [208,209,210]. Together, these data emphasise the importance of investigating not only the quantity of protein, but also the quality and balance of amino acids within proteins.

## 5. Limitations of the Single Nutrient Approach

In the sections above, we have described that consuming specific types/groups of carbohydrate, fat, and protein can lead to adverse metabolic effects. While there is broad consensus that increased intake of fructose-containing carbohydrates, saturated/LCFAs and BCAAs impairs metabolic health, there is strong disagreement in the nutrition science community about the role of different macronutrients per se on health. The polarised views are fuelled by the contradictory findings of various research studies. For example, the opinion that consuming increased amounts of carbohydrates is “toxic” for cardiometabolic health is contradicted by multiple lines of evidence [167,211,212,213,214]. Contrary to the epidemiological data from the PURE study linking carbohydrates to increased mortality [7], the habitual diets of human populations with the longest lifespans are typically high in fibre-rich carbohydrates and relatively low in protein content (Blue Zone Diets) [215,216,217,218,219,220,221]. Moreover, several other epidemiological studies have linked reduced carbohydrate intake (in combination with high protein intake) with increased mortality risk [211,212,213,214]. Data from the Atherosclerosis Risk in Communities (ARIC) study from the United States revealed a U-shaped association between mortality risk and carbohydrate consumption—with consuming 50–55% daily calories from carbohydrates being optimal for longevity [222]. Similarly, contrary to the benefits described for carbohydrate-restricted/ketogenic diets, various trials in humans have shown improved metabolic outcomes (reduced body weight and adiposity, improved lipidaemic profile and better insulin sensitivity) on high carbohydrate–low fat diets (vs. control diets) [217,223,224,225]. This has led to “fat vs. carbohydrate” debates in nutrition research [167].

The contrasting views in the literature about the metabolic effects of carbohydrate consumption are a likely consequence of the conventional “single nutrient” approach to nutrition science research [218]. This methodology focuses on the metabolic effects of consuming individual nutrients but overlooks the fact that nutrients in our diet interact to influence our health [226]. Humans do not consume individual nutrients [226]; rather, our diets contain complex mixtures of macro- and micro-nutrients, and these nutrients interact with each other to affect appetite, behavioural parameters, and physiology [218]. Therefore, to reconcile the various contradictions in the nutrition science literature, a methodology is needed that enables a holistic approach to study the effects of each nutrient as well as interaction between various nutrients on biology and health [5]. In addition, the role of protein in the health effects of various diets requires particular attention given its significance in appetite physiology and endocrine signalling [167,227].

## 6. Importance of Dietary Protein Content and the Value of a Multi-Nutrient Mixture Approach to Research in Understanding the Nutritional Basis of Metabolic Physiology

Several epidemiological studies from countries around the world have shown that, compared with carbohydrate and fat, protein consumption has remained relatively stable in recent decades, indicating tight biological regulation of protein intake [167,227]. Contrary to almost constant protein intake, the actual amount of protein in the diet has declined due to its dilution by carbohydrate, and in the more recent years, by substitution of the carbohydrate with fat [167,224,228,229,230,231]. These small changes in dietary protein content have resulted in substantial increases in total calorie intake to achieve the target protein intake (protein leverage), and this has likely been one driver of the epidemic of obesity and associated metabolic diseases [167,227]. For example, analysis of data from 2005–2006 from the National Health and Nutrition Examination Survey (NHANES) showed that a 1% increase in daily energy sourced from protein resulted in a decrease of 49 kcal/day for protein–fat substitution, and a decrease of 33 kcal/day for protein–carbohydrate substitution in normal weight subjects [232]. Moreover, analysis of NHANES 2009–2014 data and data from 13 developed countries showed that protein consistently provided ~16% of energy (regardless of demographic and lifestyle factors), while there were significantly greater variations in carbohydrate and fat intake [233]. We plotted the nutrient supply data from the Food and Agriculture Organisation Statistical Database (FAOSTAT) for 100 countries for a 1980–2013 timeframe against the adult obesity estimates from the World Health Organisation (WHO), and our analyses also showed that the prevalence of obesity significantly increased with declining dietary protein content [167]. These observations support the view that the dilution of protein in the modern-day industrialised food environments facilitate greater calorie intake and promote obesity. However, a multi-nutrient methodology is required to examine how protein–fat and protein–carbohydrate interactions further influence the impact of protein dilution.

**Figure 4:**
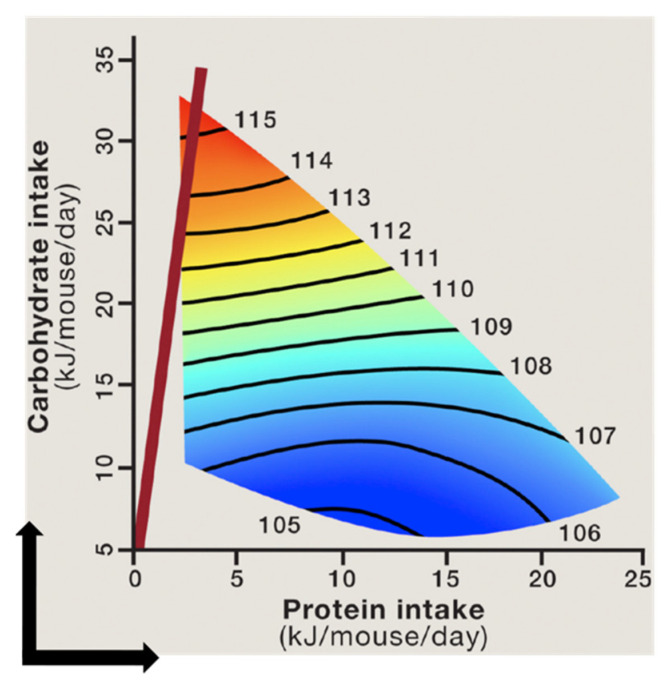
The relationship between protein and carbohydrate intake and median lifespan in mice. The lifespan remains constant along the black isolines on the surfaces, and the numbers on surfaces indicate the magnitude of lifespan (in weeks) along the isolines. The shortest lifespan is shown in blue and the longest lifespan in red. Mice fed diets with the lowest protein–carbohydrate ratio (red line) had the longest lifespan, while those fed high protein–low carbohydrate diets had the shortest lifespan [185,218] (Reprinted with permission from refs. [185,218]. Copyright 2021 Solon-Biet, SM, Simpson SJ).

The geometric framework (GF) is a nutritional modelling platform that enables an integrated assessment of the impact of nutritional composition of the diet on biological parameters [226]. In this nutritional geometry-based methodology, phenotypic responses of animals (such as body weight, adiposity, and lifespan) to diets with different compositions are plotted as response surfaces on an n-dimensional nutritional space [218]. In the case of macronutrients, the nutritional space is three-dimensional (protein, fat and carbohydrate dimension), and the response surfaces allow assessment of the effects of individual macronutrients as well as their interaction on biological outcomes [234]. The GF has been successfully employed to resolve conflicting and contradictory findings about the nutritional determinants of physiological and behavioural parameters across various species [185,208,218,234,235,236]. Importantly, the impact of macronutrient composition of diet on cardiometabolic health and longevity was investigated in a recent large-scale GF-based mouse study. Mice were maintained on 1 of 25 experimental diets with various ratios of protein, fat, and carbohydrate for their lifetime [185]. The study showed that mice fed diets that were lower in protein and higher in carbohydrate content (LPHC diets) had the longest lifespan (Figure 4). Due to protein leverage, the mice fed LPHC diets had increased food intake and body weight, but in the long term, the LPHC diets led to the best cardiometabolic outcomes (glycaemia, insulinaemia, lipidaemia, and blood pressure). On the other hand, high protein–low carbohydrate diets led to reduced lifespan and impaired cardiometabolic status despite reduced calorie intake and lower adiposity [185]. These results were in agreement with the observations in invertebrates that also showed an optimum lifespan on LPHC diets [236,237,238]. In contrast, low protein coupled with high fat yielded a phenotype of less favourable metabolic outcomes (including fatty liver) in comparison to LPHC diets [175,185]. Moreover, a low protein diet coupled with high fibre levels can reduce hyperphagia and prevent the development of insulin resistance and type 2 diabetes [239,240]. These observations clearly show that effects of consuming a particular nutrient (e.g., protein) on health and longevity is dependent on the overall nutritional composition of the diet. Mechanistically, the health benefits of LPHC diets were associated with reduced hepatic mTOR activity, increased circulating levels of metabolically beneficial FGF21 hormone, increased UCP1 expression in brown adipose tissue, and increased mitochondrial activity [185,208].

**Figure 5:**
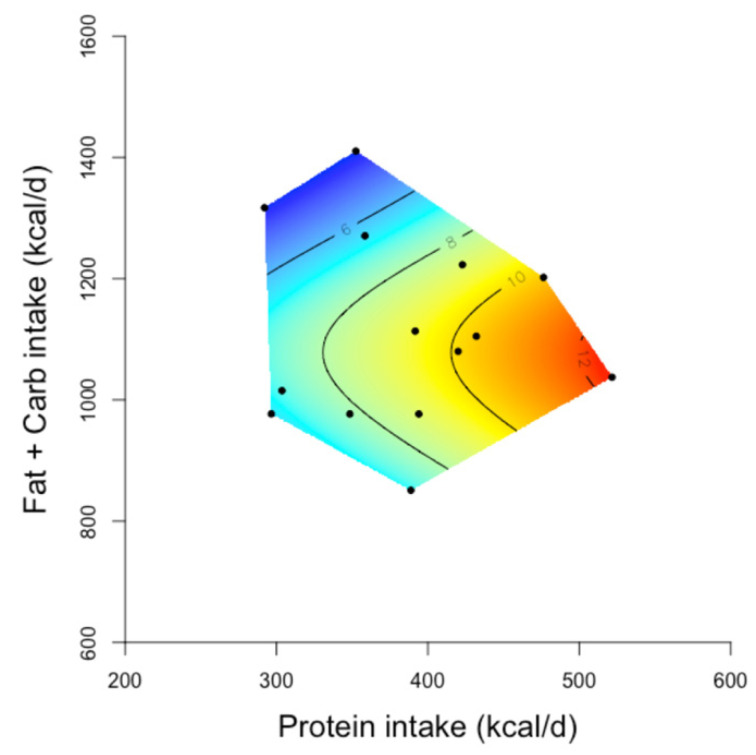
The relationship between protein and non-protein (fat and carbohydrate intake) and decrease in body weight from baseline on carbohydrate restricted/ketogenic diets [167] (Reprinted with permission from ref [167]. Copyright 2021 Wali, J.A). Two-dimensional geometric framework (GF) surfaces showing the relationship between the intake of energy from protein and non-protein (fat and carbohydrate) sources and the decline in body weight (kg) in study participants. In the GF surfaces, red colour shows the maximum and blue colour shows the minimum decrease in body weight, and black dots represents the intake of protein and non-protein energy reported in each study. Human studies of 2.5–6 months duration which reported the average daily intake of macronutrients of the study participants on carbohydrate-restricted diets and their mean decrease in body weight (*n* = 14 studies) achieved on these diets (vs. baseline measurements) were included in these GF plots. The decrease in body weight became greater as the protein intake increased (red areas of the surface). Adapted with permission of the authors Wali et al. 2020 [167].

We recently analysed data from human trials of ad libitum carbohydrate-restricted/ketogenic diets of 2.5–6 months duration using GF. It was found that compared with habitual diets, the decrease in daily calorie intake and weight loss was a function of protein consumption. The decrease in calorie intake and loss of body weight became more pronounced with increasing proportions of daily calories from protein (Figure 5) [167]. In addition, analysis of epidemiological data from recent decades for >100 countries across the globe showed that, in early life, the mortality was minimal if 40–45% of energy was obtained from fat and carbohydrate and 16% from protein. However, in later life, increasing carbohydrate to around 65% and reducing protein to 11% led to the lowest level of mortality [241]. Together, these observations in animals and humans highlight the value of GF in revealing the nutritional basis of metabolic health and disease.

## 7. Conclusions

Increased intake of all three macronutrients (protein, fat, and carbohydrate) has been associated with insulin resistance and metabolic disease in the literature. There is broad consensus that specific subtypes of carbohydrate (fructose-containing carbohydrates), fat (saturated LCFAs), and protein (BCAAs) are harmful for metabolic health [6,7,8,9,10,11,12,13,14,15,16]. Fructose is thought to induce insulin resistance by stimulating DNL, reducing fat oxidation and increasing uric acid production [6,43,105,106,115]. LCFAs likely cause insulin resistance by promoting the accumulation of toxic lipid species such as ceramide and DAG in insulin-sensitive tissues and by increasing ROS production [141,142,146]. BCAAs are thought to activate mTOR and inhibit insulin signalling by enabling insulin-induced IRS-1 degradation [207]. However, at the level of overall macronutrient groups, there is disagreement in the literature if increased consumption of carbohydrate, protein, or fat is detrimental or beneficial for metabolic health. An example of this disagreement is the carbohydrate vs. fat debate in the nutrition science community [167]. These inconsistencies arise from the inherent limitations of the single nutrient approach that has traditionally been used in nutrition research [218]. The nutrients in our diet interact to influence metabolic outcomes, and a multi-nutrient approach is therefore needed to reconcile various controversies about the effects of consuming different nutrients [226]. The geometric framework (GF) provides such a platform for an integrated assessment of the effects of consuming different nutrients on health and disease [226]. Studies in animals employing the GF platform have shown that low protein–high carbohydrate diets are optimal for increased life- and health-span [185,208,218,234,235,236]. Moving forward, animal studies and human trials based on the GF methodology are needed for a comprehensive understanding of the impact of interaction between protein, fat, and carbohydrate on insulin and metabolic signalling and their impairment in response to an unhealthy diet.

## Data Availability

Not applicable.
